# Multi-omics staging of locally advanced rectal cancer predicts treatment response: a pilot study

**DOI:** 10.1007/s11547-024-01811-0

**Published:** 2024-03-27

**Authors:** Ilaria Cicalini, Antonio Maria Chiarelli, Piero Chiacchiaretta, David Perpetuini, Consuelo Rosa, Domenico Mastrodicasa, Martina d’Annibale, Stefano Trebeschi, Francesco Lorenzo Serafini, Giulio Cocco, Marco Narciso, Antonio Corvino, Sebastiano Cinalli, Domenico Genovesi, Paola Lanuti, Silvia Valentinuzzi, Damiana Pieragostino, Davide Brocco, Regina G. H. Beets-Tan, Nicola Tinari, Stefano L. Sensi, Liborio Stuppia, Piero Del Boccio, Massimo Caulo, Andrea Delli Pizzi

**Affiliations:** 1 Center for Advanced Studies and Technology (CAST), University “G. d’Annunzio” of Chieti-Pescara, Chieti, Italy; 2 Department of Innovative Technologies in Medicine and Odontoiatry, “G. d’Annunzio” University, Chieti, Italy; 3 Department of Neuroscience, Imaging and Clinical Sciences, “G. d’Annunzio” University, Chieti, Italy; 4 Department of Radiology, Stanford University School of Medicine, Stanford, CA USA; 5 Department of Radiology, SS. Annunziata Hospital, “G. d’Annunzio” University, Via dei Vestini, 66100 ChietiChieti, Italy; 6 Department of Radiology, Netherlands Cancer Institute, Amsterdam, The Netherlands; 7 Unit of Ultrasound in Internal Medicine, Department of Medicine and Science of Aging, “G. D’Annunzio” University, Chieti, Italy; 8 Medical, Movement and Wellbeing Sciences Department, Via Medina 40, 80133 Naples, Italy; 9 Division of Pathology, ASST of Valtellina and Alto Lario, Sondrio, Italy; 10 Department of Medical, Oral and Biotechnological Sciences and CeSI-MeT, “G. D’Annunzio” University of Chieti, Via dei Vestini, 66100 Chieti, Italy; 11 Department of Medicine and Aging Science, “G. D’Annunzio” University of Chieti, Via dei Vestini, 66100 Chieti, Italy; 12 Department of Pharmacy, “G. D’Annunzio” University of Chieti, Via dei Vestini, 66100 Chieti, Italy; 13 Clinical Oncology Unit, SS. Annunziata Hospital, Via dei Vestini, 66100 Chieti, Italy; 14 GROW School for Oncology and Developmental Biology, Maastricht University, Maastricht, The Netherlands; 15 Department of Psychological, Health and Territory Sciences, “G. d’Annunzio” University of Chieti-Pescara, 66100 Chieti, Italy

**Keywords:** Rectal cancer, Treatment response, Magnetic resonance imaging, Multi-omics, Radiomics, Metabolomics

## Abstract

Treatment response assessment of rectal cancer patients is a critical component of personalized cancer care and it allows to identify suitable candidates for organ-preserving strategies. This pilot study employed a novel multi-omics approach combining MRI-based radiomic features and untargeted metabolomics to infer treatment response at staging. The metabolic signature highlighted how tumor cell viability is predictively down-regulated, while the response to oxidative stress was up-regulated in responder patients, showing significantly reduced oxoproline values at baseline compared to non-responder patients (*p*-value < 10^–4^). Tumors with a high degree of texture homogeneity, as assessed by radiomics, were more likely to achieve a major pathological response (*p*-value < 10^–3^). A machine learning classifier was implemented to summarize the multi-omics information and discriminate responders and non-responders. Combining all available radiomic and metabolomic features, the classifier delivered an AUC of 0.864 (± 0.083, *p*-value < 10^–3^) with a best-point sensitivity of 90.9% and a specificity of 81.8%. Our results suggest that a multi-omics approach, integrating radiomics and metabolomic data, can enhance the predictive value of standard MRI and could help to avoid unnecessary surgical treatments and their associated long-term complications.

**Supplementary Information:**

The online version contains supplementary material available at 10.1007/s11547-024-01811-0.

## Introduction

Colorectal cancer (CRC) represents the third most frequent cancer in the world and the fourth cause of cancer death [1]. Currently, the total mesorectal excision (TME) after neoadjuvant chemo-radiotherapy represents the standard of care for patients with locally advanced rectal cancer (LARC) (≥ T3 or *N* +) is the total mesorectal excision (TME) after neoadjuvant chemo-radiotherapy (CRT)^2^. Watchful waiting programs allow patients with complete response following CRT (“responders”) to be closely monitored via imaging and endoscopy, opting for organ-preserving treatment strategies; however, these programs are adopted only in selected institutions [2]. LARC receiving neoadjuvant CRT showed complete response rates ranging from 5 to 44% [3]. Recently, there has been a growing interest in new biomarkers of CRT response to improve patients’ selection for more intensive therapy or watchful waiting.

The gold standard method for staging rectal cancer and evaluating the response to treatment is magnetic resonance imaging (MRI) [4]. Moreover, MRI plays a crucial role in patient selection and monitoring in the watchful waiting strategy [5]. Indeed, MRI demonstrates remarkable precision in qualitatively evaluating prognostic factors, such as tumor location, extent, the distance from the anal sphincter complex, presence of mesorectal fascia infiltration and extramural vascular invasion [6]. In a recent multi-reader study comparing four MRI methods for rectal tumor response evaluation, the average accuracy ranged between 62 and 68% and improved when diffusion-weighted imaging (DWI) was included [7].

Recently, multi-omics approaches investigating CRC at molecular level have shown potential to improve diagnosis and prognostic assessment of colon cancer [8, 9]. Among these “omics” disciplines, “radiomics” and “metabolomics” recently showed the potential predictive role in tumor response assessment [10–13]. The term “radiomics” refers to the application of information engineering approaches to radiologic images delivering a large number of image features related to the shape, intensity and texture heterogeneity within a given volume of interest, otherwise invisible to the naked eye. Tumors are heterogeneous at the genetic and histopathological level, and high intratumoral heterogeneity is associated with a poor prognosis [14]. This approach can overcome the limit of random sampling or biopsy that does not allow for a complete assessment of the phenotype or genetic heterogeneity within a tumor [14]. Based on these characteristics and thanks to the continuous technological improvement, the radiomics approach has been used for the preoperative assessment of treatment response [15–18]. Metabolomics, by studying and quantifying the metabolites present in biological fluids, offers an instant view of the system, providing useful information for understanding the processes taking place in the analyzed organism. Tumor growth follows a variety of metabolic pathways resulting in the accumulation of specific intermediate metabolites and metabolomics represent a growing discipline in the characterization of serum metabolites in cancer [19, 20] can be used as prognostic markers for response to CRT. [21–25]. To date, integrating “-omics” disciplines may represent a new framework for personalized cancer care.

This study presents a cutting-edge multi-omics approach combining MRI-based radiomics features with untargeted metabolomics data, with the aim of developing an experimental workflow capable of predicting the treatment response in patients diagnosed with LARC. This novel noninvasive method called “radiometabolomics” may pave the way to further larger studies improving the role of “omics” disciplines in rectal cancer.

## Methods

### Subjects

**Fig. 1:**
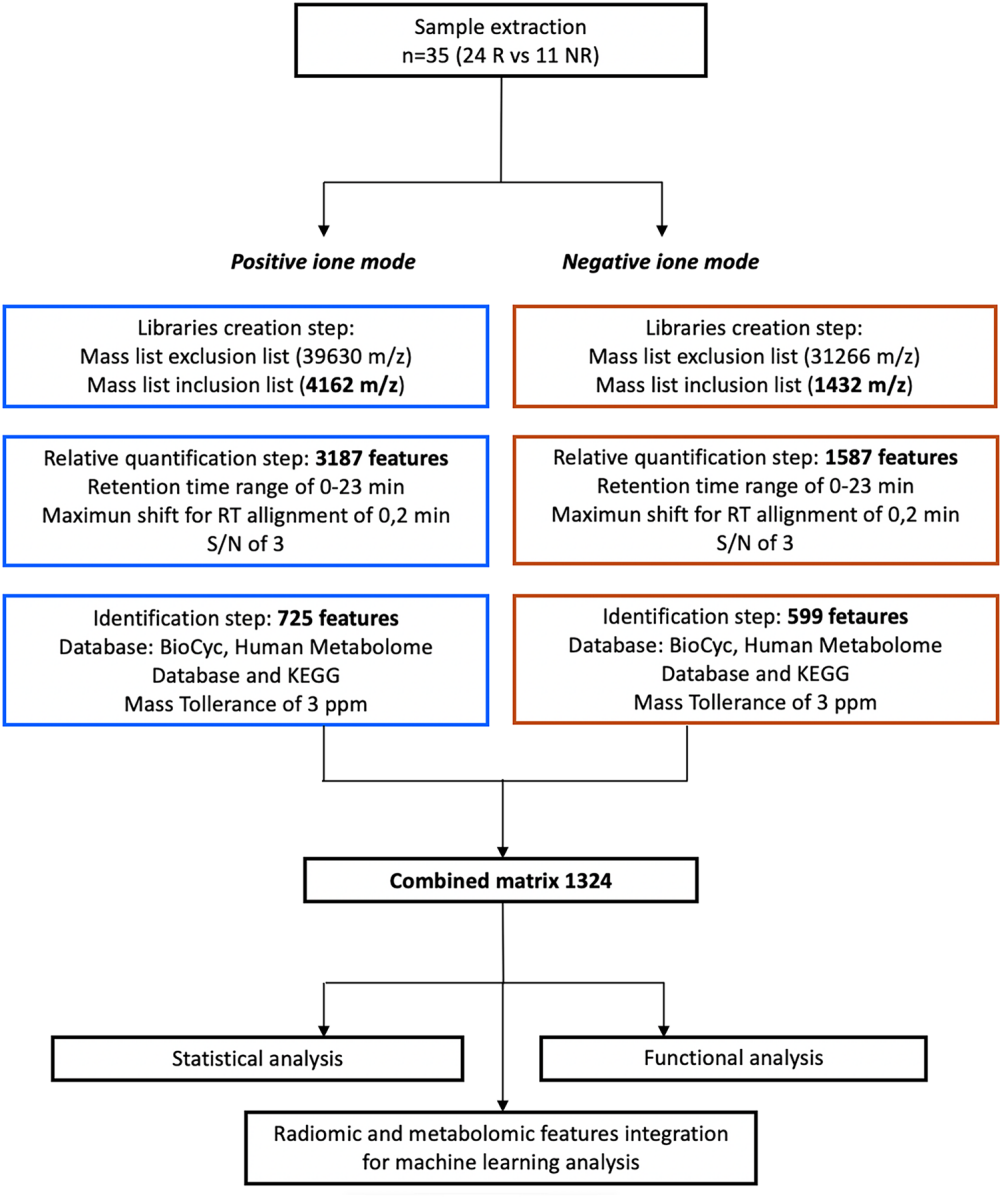
Workflow of untargeted metabolomics approach. The number of features taken into consideration in the various steps, up to the final data matrix, is shown in bold. Each processing step was described detailing the inclusion and exclusion criteria. Exclusion list includes compounds present in the blank sample that are considered potential contaminants. Inclusion list includes molecules present in the sample excluding contaminants. Chromatographic retention times (RT) and the minimum signal-to-noise ratio (*S*/*N*) were the inclusion criteria for the quantification step. The databases and the mass tolerance (expressed in ppm) were the inclusion criteria in the identification step

This retrospective study was approved by the local Ethical Committee. A total of 140 consecutive patients who underwent rectal MRI for tumor staging between February 2013 and February 2019 were included (Fig. 1). Inclusion criteria were (1) biopsy-confirmed non-mucinous LARC, (2) 3.0 T MRI examination, (3) clinical outcome assessment on surgical specimen (complete or not complete response), (4) long-course CRT and (5) availability of metabolomics data. In total, 105 patients were excluded: 12 were mucinous cancers, 33 were treated in other centers, and the final clinical outcome was not available, 18 were considered unfit for long-course CRT due to poor clinical conditions stat, 5 patients had severe MR susceptibility artifacts in the pelvis (hip replacement), and 37 had no metabolomics data. The study population was composed of 35 patients. Descriptive baseline characteristics of included patients are detailed in Table 1. The study workflow is shown in Fig. 5.

**Table 1:** Descriptive baseline characteristics of included patients (*n* = 35)

Variable	Value
Gender	
Male	22 (63%)
Female	13 (37%)
Median age (IQR*)	66 (63–74)
MRI examination	35
Staging MRI Features	
Location	
High	2 (6%)
Middle	17 (48%)
Low	16 (46%)
Craniocaudal Extension, mm (Mean ± SD)	47 ± 20
Distance from IAS, mm (Mean ± SD)	31 ± 26
Depth of Extramural Invasion, mm (Mean ± SD)	7 ± 7
Mesorectal Fascia Infiltration	11 (31%)
Extramural Vascular Invasion	21 (60%)
Primary cT stage**	
T1–T2	2 (6%)
T3	30 (86%)
T4	3 (8%)
Primary cN stage**	
N0	7 (20%)
N1	18 (51%)
N2	10 (29%)
Treatment response***	
Responders	24 (69%)
	16 TRG1 (46%)
	8 TRG2 (23%)
Non-responders	11 (31%)
	8 TRG3 (23%)
	3 TRG4 (8%)

^*^ = IQR Inter-quartile range; SD standard deviation; IAS internal anal sphincter; EMVI extramural vascular invasion

^**^Assessed with MRI and derived from clinical MRI reports in the hospital’s patient database

^***^Assessed according to Mandard Tumor Regression Grade (TRG) system on surgical specimen after neoadjuvant treatment

### Chemo-radiotherapy

Long-course radiotherapy consisted of 3D conformal technique. A total dose of 4500 cGy (180 cGy/day) was delivered to the pelvic nodes. It was followed by a sequential boost of 540 cGy (180 cGy/day; total dose 5040 cGy) or a concomitant boost of 1000 cGy (100 cGy/day, 2 times/week; total dose 5500 cGy). It was firstly used a 3D-CRT technique and then a simultaneous integrated boost with intensity-modulated radiotherapy (220 cGy/day, total dose 5500 cGy). 5-Fluorouracil and leucovorin or capecitabine were administered in different schedules as concomitant chemotherapy.

### MRI protocol

A 3 T MRI (Achieva, Philips Medical System, Best, the Netherlands) equipped with a phased array surface coil was used for the rectal study. The MRI protocol included T2w and DWI images acquired with a plane perpendicular to the tumor major axis. ADC maps were calculated for each patient. Detailed information about the MRI protocol has been previously documented and is accessible in the supplementary material (Appendix 1) [6, 15, 26].

### Imaging analysis

Two independent whole-volume tumor segmentations for each patient were manually performed by two readers on T2w and ADC. One reader was a board-certified radiologist with 5 years of expertise in rectal MRI and the other was a senior radiology resident. The tumor presence was defined as intermediate signal intensity on T2w images and a corresponding hypointensity on ADC [4]. All the segmentations were used as masks for the following analysis. An open-source computing platform, 3DSlicer Version 4.8 (www.3dslicer.org), was used for image segmentation [15, 27].

### Staging MRI features

Four weeks after the manual segmentation, the two readers evaluated in consensus eight staging MRI-based features (sMRI) on the T2w images. These features regarded tumor location, craniocaudal extension, distance from the internal anal sphincter, mesorectal fascia infiltration, extramural vascular invasion, extramural depth of tumor invasion, T-stage and N-stage [15].

### Radiomic features

An open-source platform, PyRadiomics, guided the extraction of radiomics features from the segmentations of the two readers [28]. A reproducibility assessment was performed. Both the MR images and the segmentations were resampled using isotropic voxel dimensions of 1 × 1 × 1 mm to avoid data heterogeneity bias and to ensure reproducibility. Ten built-in filters (original, wavelet, Laplacian of Gaussian (LoG), square, square root, logarithm, exponential, gradient, LBP2D and LBP3D) were utilized to process the data, resulting in the calculation of seven feature classes (first-order statistics, shape descriptors, glcm, glrlm, ngtdm, gldm and glszm).

### Blood sample extraction and processing

60 μL of serum was extracted with 180 μL of methanol on ice, after 30 min of incubation on ice, the sample was centrifugated at + 4 °C for 30 min, the supernatant was dried in speedvac and the dry residue was resuspended in 120 μL by using a mixture of water and acetonitrile (ACN) 30:70 for metabolic analyzes.

### Untargeted metabolomics analysis

Ten μL of extracted metabolites was analyzed in triplicate by LC–MS/MS with a Dionex UltiMate 3000 RSLCnano System (Thermo Fisher Scientific) coupled to an Orbitrap Fusion Tribrid mass spectrometer (Thermo Fisher Scientific), using the Deep Scan AcquireX data dependent acquisition tool. Metabolites were separated on an Accucore™ C18 (2.1 mm I.D., 150 mm L., 2.6 μm ps, Thermo Fisher Scientific) HPLC column. The flow rate was set to 300 µL/min with a total run time of 30 min and the following chromatographic gradient (mobile phase A: 0.1% formic acid (FA) in water (H_2_O); mobile phase B: 0.1% FA in ACN: from 5 to 70% of B in 13.5 min followed by 70 to 98% in 2 min, maintaining 98% B for 7 min, from 98 to 5% B in 0.5 min and maintaining 5% B until the end of the run). The mass spectrometer, providing a 60,000 resolution in full scan mode throughout the mass range, was equipped with a H-ESI spray source. The acquisition was achieved in both positive and negative ion polarity, using stepped HCD fragmentation (collision energies: 20, 40 and 120%) and Orbitrap for MS^2^, fixed collision-induced dissociation (CID) fragmentation (collision energy: 35%) and ion trap for MS^3^. Precursor ions in the range 160 to 2000 m/z with an absolute intensity above 5.0 × 10^2^ were selected for MS^2^, while fragment ions above the threshold of 8.0 × 10^3^ were chosen for MS^3^. Deep Scan AcquireX data dependent acquisition tool was used by analyzing a representative pool sample for the creation of excluded and included mass list in both MS scan acquisition mode and MS^2^ and MS^3^ for the identification step.

### Metabolomics feature extraction

Raw data were processed using ChemSpider and mzCloud to search databases of MS^1^ and fragmentation scans, respectively, in Compound Discoverer version 3.1 (Thermo Fisher Scientific). Mass lists of “Endogenous Metabolites Database 4400 Compounds” and “Extractables and Leachables HRAM Compound Database” were used as well, mostly to recognize contaminants. Metabolite identification was based on accurate mass and mass fragmentation pattern spectra, but annotation required at least in part a manual contribution after applying the FISh scoring. A mass tolerance of 3 ppm was set for feature matching, and the log2 fold change was used to compare the differential abundance of compounds in each sample.

### Functional analysis of metabolomics signature

Metabolites ratios were uploaded for “Core Analysis” through Ingenuity Pathway Analysis (IPA software, Qiagen, Hilden, Germany) to statistically map the modulated molecules for their functional annotations in terms of canonical pathways, upstream regulators analysis and downstream effects networks. Molecular pathways and predicted upstream regulators with overlap *p*-value < 0.05 and activation z-score > 2 or < − 2 were considered as meaningful on both statistical and biological points of view.

### Machine learning: kNN-based classification

Machine learning (ML) approaches aim to exploit data multi-dimensionality to identify statistical dependences among variables for predictive, modeling and classification purposes. In this study, the k-nearest neighbors (kNN) classifier was employed to classify the responder and non-responder groups [29]. The kNN classifier labels a new instance based on the majority class of the k closest instances from the training data. The kNN algorithm commences by receiving an instance whose class label is unknown. Using a distance metric, such as Euclidean distance, it then computes the distance between this new instance and every instance in the training set. Next, it selects the k instances from the training set that are closest to the new instance based on their distance from the new instance. The new instance is then tagged with the class label that is most prevalent among its k-nearest neighbors (“majority vote”). *k* is a user-specified parameter that can be selected by cross-validation or other methods. Larger values of k result in a smoother decision boundary, while smaller values of k result in a more complex decision boundary. One of the benefits of kNN is that it is simple and easy to understand. However, it can be computationally expensive, particularly when the size of the training set size is large [13]. sMRI features, radiomic features (ADC and T2w evaluated by two operators) and metabolomic data (MD) were considered as input of the machinery, both independently and together, thus allowing to develop five different models relying on, respectively, sMRI only, MD only, ADC only, T2w only and sMRI + MD + ADC + T2w. The classifier’s input data were normalized (z-score), whereas the output was defined labeling the non-responder patients as class “0” and the responder participants as class “1.” The Euclidean distance was employed as the distance metric, the weights assigned to the k-nearest neighbors were the inverse of the squared distance, and the number of neighbors was considered as an hyperparameter of the model and it was optimized within the nested cross-validation framework [30–32].

To mitigate the poor generalization performances potentially deriving from the large number of features with respect to samples, feature selection procedures were employed [33]. Feature selection was performed in two steps. Firstly, a subset of the available features was selected considering only those features with a high “inter-observer” reliability, i.e., only those robust to differences introduced by the manual identification of the region of interest by the independent radiologists. Radiomics features with high inter-observer correlation (*r* > 0.95) were considered reliable and used for further analysis [34, 35]. Of note, such feature selection was performed outside the nested cross-validation. In fact, this feature selection does not exploit data output label and does not create classification overfitting. The second step of feature selection was performed implementing a wrapper method [36, 37]. In this study, a sequential forward selection was employed: It started with no feature in the model, and in each iteration, a feature was added to produce the highest increase in performance until the addition of a new variable did not improve the model’s performance. Differently to the first step of feature selection, the wrapper method exploits the output labels and creates data overfitting without samples separation in train and test sets. Hence, similarly to the hyperparameter optimization of the kNN classifiers, the wrapper approach was iterated within the nested cross-validation (nCV).

To optimize hyperparameters and ensure the model’s generalizability without overfitting, three distinct datasets are necessary. Firstly, a training set is used to train the model with various hyperparameter values. Secondly, a validation set is employed to select the best hyperparameters based on performance. Finally, a test set is used to evaluate the final model’s performance. This approach allows for effective hyperparameter tuning and unbiased assessment of the model’s performance on unseen data. However, when a reduced sample numerosity is available, this data separation might drastically reduce the training sample, making it difficult for the data-driven model to be properly fitted. The nCV is an extension of this method that helps to reduce the impact of sample loss across multiple sets while avoiding results biases and data overfitting [30–32]. In detail, data are separated into folds, and the model is trained iteratively in a nested way on all but onefold of the data. The inner loop identifies the optimal hyperparameters (validation, in our case it identified also features employing the wrapper method) while the outer loop estimates how well the model performs over iterations (test). The approach is described as leave-one-out nCV if the number of folds equals the number of samples (onefold per sample) [38, 39]. This method is ideal for medical settings because each sample corresponds to a single patient. In this study, a leave-one-out nCV was used to train, validate and test the kNN model. To define the optimal model hyperparameter and identify the selected features reported in the manuscript, a majority voting algorithm was employed across the cross-validation iterations. Since the study sample provided non-balanced classes, an iterative procedure (1000 iterations) was performed to train and test the machinery with balanced classes via random subsampling of the larger class at each iteration. The ML analysis was implemented in MATLAB (MATLAB 2021b©, Mathworks Natick, MA, USA).

#### Reference standard

For all patients, a Tumor Regression Grade (TRG) 1 or 2, assessed according to Mandard’s classification, was considered as major pathological response [40–42].

In detail, TRG1 and TRG2 were labeled as responders (R) while TRG3 and TRG4 as non-responders (NR) [43].

#### Statistical analysis

The classification performance was assessed by evaluating the confusion matrix associated with the out-of-training samples, which provides information about the sensitivity and specificity of the classifier. In addition, the receiver operating characteristic (ROC) analysis was employed, considering the area under the ROC curve (AUC) as indicative of the classification performance of each proposed model. Since a bootstrap procedure was implemented to balance the classes randomly, the average AUC and the standard deviation of the distribution delivered by the procedure are reported. The *p*-values associated with the average AUCs (probability of obtaining an AUC value above 0.5 by chance) and that associated with average AUCs comparisons (probability of two AUCs being generated from the same underlying distribution) were evaluated generating a metrics’ confidence interval through a bootstrapping approach (1000 iterations). The statistical analysis was performed in MATLAB (MATLAB 2021b©, Mathworks Natick, MA, USA). PLS-DA and volcano plot for metabolomics data were performed by the online tool Metaboanalyst (https://www.metaboanalyst.ca/). Violin plot were performed with BoxPlotR online tool, and correlation and Heatmaps were performed with GraphPad Prism 7.

## Results

### Staging MRI features

Nine MRI staging features (sMRI) were evaluated by radiologists (refer to the method section for further information). Of the nine features, the only feature that was different on average between the 24 responders (*R*) and 11 non-responders (NR) was the mesorectal fascia infiltration (*p* < 0.05).

### Radiomics

In total, 1470 features were extracted with PyRadiomics for each subject, image type and reader. Of these 1470 features, 919 for apparent diffusion coefficient (ADC) and 916 for T2-weighted (T2w) images were highly reproducible (*r* > 0.95) when computed within the masks identified by the two radiologists. The reproducible features were averaged between the two readers and were used for further analysis. Of the selected features, 109 for the ADC and 124 for the T2w images showed significant average differences between the 24 R and 11 NR (*p* < 0.05, uncorrected).

### Metabolomics

Sera from 24 R and 11 NR were analyzed using an untargeted metabolomics approach, to identify and relatively quantify as many serum metabolites as possible. Following the workflow shown in Fig. 1, a representative pool of blood samples was used to build a library of mass spectra and fragmentation mass spectra for the metabolite identification step, in both positive and negative acquisition mode. In this phase of library construction, the triplicate analysis of a blank sample allowed us to create a mass exclusion list composed of 39,630 m/z and 31,266 m/z for acquisition in positive and negative mode, respectively. At the same time, the analysis of a representative sample allowed us to create an inclusion list of species to be quantified and identified in the next step on the samples individually (4162 and 1432 species in positive and negative mode, respectively), as summarized in the workflow in Fig. 1. In the following step, samples from 35 patients were individually analyzed in mass scanning in both positive and negative acquisition mode, to obtain a chromatographic profile for the metabolite’s relative quantification step. In total, 3187 and 1587 features were quantified in positive and negative modes, based on the following quantification parameters: Retention time (RT) 0–23 min, maximum shift for allign RT = 0.2 min and signal-to-noise ratio *S*/*N* = 3.

Of these, 1,324 features were identified as specific metabolites by matching with the library of the acquired fragmentation mass spectra. The databases used for identification were BioCyc, Human Metabolome Database and KEGG, considering a mass tolerance of 3 ppm.

**Fig. 2:**
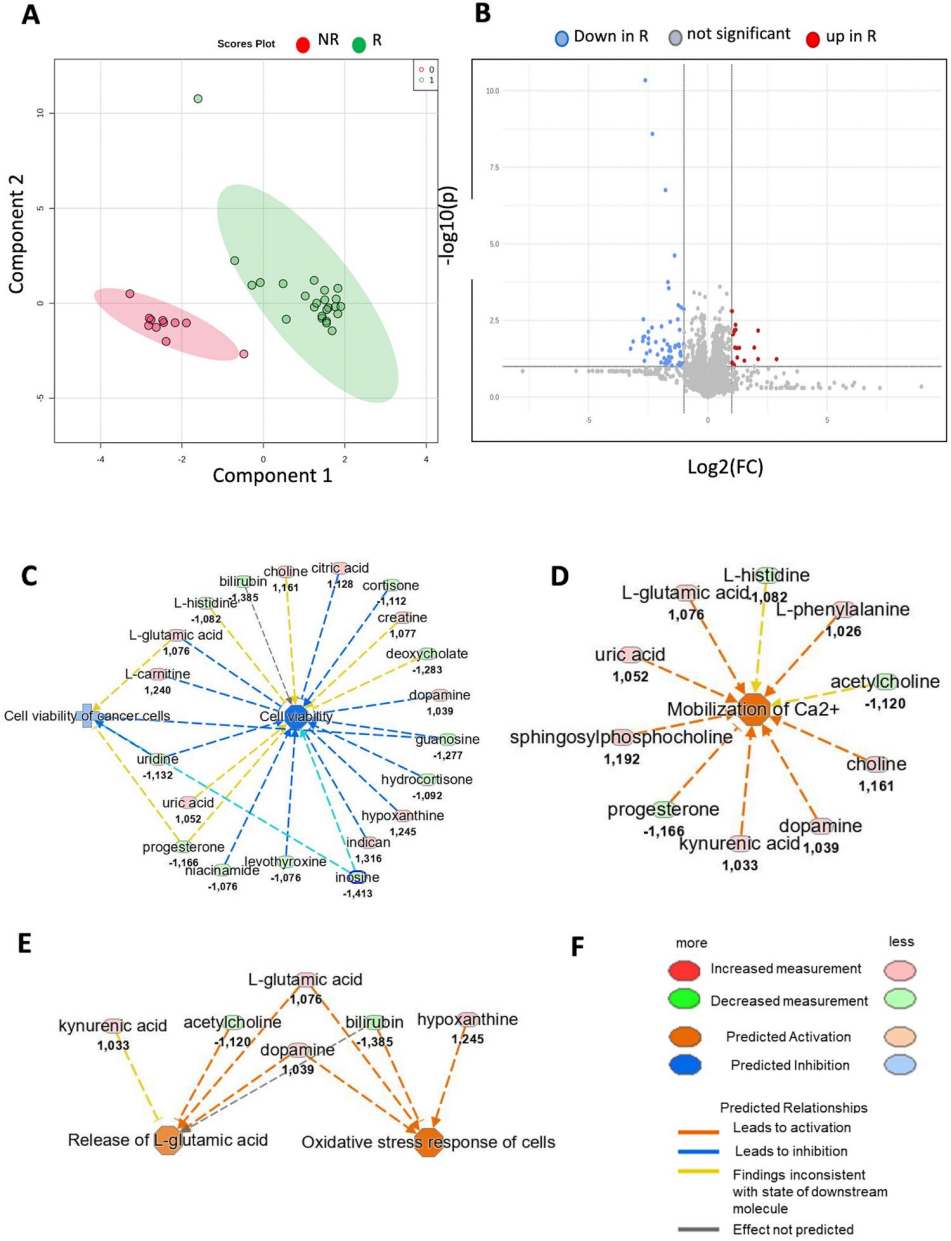
**A** sPLS-DA based on 4000 features in the plasma of responder (R) and non-responder (NR) patients. **B** Volcano plot of 4000 metabolomic features classifying them in not significant (gray), significantly down-regulated in RP (blue dots) and significantly up-regulated in RP (red dots). **C** Prediction of down-regulated cellular functions “Cell viability” and “Cell viability of cancer cells” in R patients. **D** Predicted of up-regulated cellular function “Mobilization of Ca2 + ” in R patients. **E** Predicted up-regulation of cellular function “Oxidative stress response of cells” and “Release of l-glutamic acid” in R patients. **F** Legend of color code for increased and decreased measurement of metabolites, and predicted activation and inhibition of disease and cellular function

The identified metabolites were used for statistical analysis and functional analysis through the Ingenuity Pathway Analysis (IPA software, Qiagen, Hilden, Germany) bioinformatics tool and machine learning analysis. The sparse partial least-squares discriminant analysis (sPLS-DA) algorithm was applied as exploratory multivariate statistical analysis (Fig. 2A), indicating that metabolomics serum signatures could distinguish R from NR patients at T0. Univariate statistical analysis revealed 75 compounds significantly different when comparing R and NR at T0, as shown in the volcano plot in Fig. 2B. IPA functional investigation of the endogenous compounds identified, quantified and mapped in the Kyoto Encyclopedia of Genes and Genomes (KEGG) and Human Metabolome Database (HMDB), highlighted a predicted down-regulation of “Cell viability” and “Cell viability of cancer cells” (*p*-value = 2.09 E10^−3^; z-score = − 1.86) biological function in R patients compared to NR patients, before CRT (Fig. 2C). Moreover, in R patients, IPA investigation highlighted a predicted significant up-regulation of the following cellular functions: “Mobilization of Ca2 + ” (*p*-value = 1.74 E10^−5^; *z*-score =  + 2.17), “Oxidative stress response of cells” (*p*-value = 2.97 E10^−4^; z-score =  + 1.98) and “Release of L-glutamic acid” (*p*-value = 6.15 E10^−5^; z-score =  + 1.17) (Fig. 2D–E).

### Multi-omics kNN-based classification

**Fig. 3:**
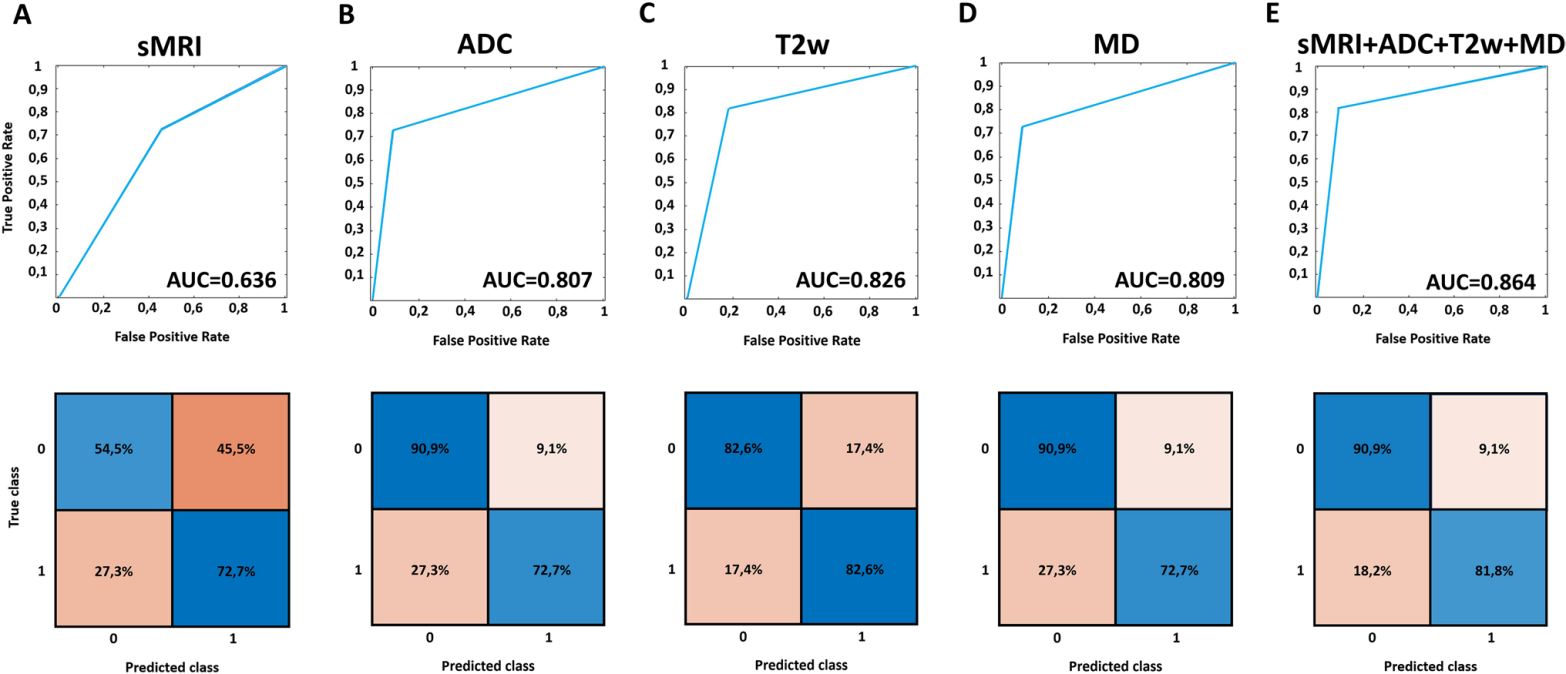
ROC curve and best cutoff point confusion matrix delivered by the classification performed relying on **A** sMRI data, **B** ADC metrics, **C** T2w metrics, **D** MD data and **E** sMRI + ADC + T2w + MD data

When using the k-Nearest Neighbors (kNN) classifier on sMRI metrics, only the Mesorectal Fascia Infiltration was selected via the wrapper procedure. The classification performance evaluation delivered an out-of-training sample ROC with an AUC of 0.636 (± 0.094, *p*-value = 0.10). The optimized cutoff point delivered an out-of-training sample sensitivity of 54.5% and an out-of-training sample specificity of 72.7%. In Fig. 3A, ROC curve is presented together with the associated confusion matrix for the optimized cutoff point.

When using metrics extracted from apparent diffusion coefficient (ADC) maps, the “original_shape_Maximum2DDiameterSlice” and “wavelet_LLH_glrlm_RunLengthNonUniformity” features were selected. The machine learning (ML) framework achieved ROC performance with an AUC of 0.807 (± 0.097, *p*-value = 0.001) and a best sensitivity of 90.9% coupled with a specificity of 72.7% (Fig. 3B).

As concerns the T2w analysis, 4 features were selected: exponential_firstorder_Energy, “squareroot_glcm_InverseVariance,” “wavelet_LLH_glszm_SizeZoneNonUniformity” and “lbp_3D_m1_glrlm_RunLengthNonUniformity.” An AUC of 0.826 (± 0.097, *p*-value = 0.0007) coupled with a best-point sensitivity and a specificity of 82.6% were obtained (Fig. 3C).

Feature selection on metabolomic data (MD) identified 2 salient compounds, oxoproline and proline. The classification delivered an AUC = 0.809 (± 0.041, *p*-value = 0.001) with best sensitivity of 90.9% and specificity of 72.7% (Fig. 3D).

When considering all the available features (i.e., sMRI + ADC + T2w + MD), 4 features were identified as the most predictive by the wrapper procedure: “wavelet_LLH_glrlm_RunLengthNonUniformity” and “gradient_glszm_GrayLevelNonUniformity” from ADC images, “wavelet_HHH_glcm_DifferenceAverage” from T2w images and oxoproline from MD data. The AUC obtained was 0.864 (± 0.083, *p*-value = 0.0002) with a best-point sensitivity of 90.9% and a specificity of 81.8% (Fig. 3E). The performances of the investigated models are reported in Table 2. Notably, when comparing the AUC of the different models, statistically significant differences were found when comparing sMRI-based classification with the multi-omics classification (Tables 3 and 4).

**Table 2:** Classification performances of the proposed models expressed as AUC, sensitivity and specificity

Technique	AUC	Sensitivity (%)	Specificity (%)	Selected features
sMRI	0.636	54.5	72.7	Mesorectal Fascia Infiltration
MD	0.809	90.9	72.7	Oxoproline Proline
ADC	0.807	90.9	72.7	original_shape_Maximum2DDiameterSlice wavelet_LLH_glrlm_RunLengthNonUniformity
T2w	0.826	82.6	82.6	exponential_firstorder_Energy squareroot_glcm_InverseVariance wavelet_HHL_glszm_SizeZoneNonUniformity lbp_3D_m1_glrlm_RunLengthNonUniformity
MD + ADC + T2w	0.864	90.9	81.8	ADC: wavelet_LLH_glrlm_RunLengthNonUniformity gradient_glszm_GrayLevelNonUniformity T2w: wavelet_HHH_glcm_DifferenceAverage MD: Oxoproline

**Table 3:** Comparison between the AUCs of the developed models

Comparisons	z-stat	*p*-value
MD vs. ADC	0.019	0.985
MD vs. T2w	− 0.101	0.867
ADC vs. T2w	− 0.187	0.851
MD + ADC + T2w vs. MD	0.577	0.564
MD + ADC + T2w vs. ADC	0.596	0.551
MD + ADC + T2w vs. T2w	0.410	0.682
sMRI vs. MD	− 1.411	0.158
sMRI vs. ADC	− 1.392	0.164
sMRI vs. T2w	− 1.573	0.115
sMRI vs. MD + ADC + T2w	− 1.970	0.048

**Table 4:** Statistical test (Chi-square stat and t-stat) between the two groups (non-responders vs. responders) for the metrics identified by the feature selection procedures

	Metric	Chi-square stat	*p*-value
sMRI	mesorectal fascia infiltration	3.978	0.046

	Metric	t-stat	*p*-value
MD	Proline	− 1.173	0.249
	4-Oxoproline	4.910	2.40 × 10^–5^
ADC	original_shape_Maximum2DDiameterSlice	3.386	0.002
	wavelet_LLH_glrlm_RunLengthNonUniformity	3.704	7.73 × 10^–4^
	squareroot_glszm_LargeAreaLowGrayLevelEmphasis	− 0.689	0.495
	wavelet_HHL_firstorder_Energy	2.695	0.011
T2w	exponential_firstorder_Energy	2.155	0.039
	squareroot_glcm_InverseVariance	2.345	0.025
	wavelet_HHL_glszm_SizeZoneNonUniformity	0.725	0.473
	lbp_3D_m1_glrlm_RunLengthNonUniformity	4.208	1.85 × 10^–4^
	wavelet_HHH_glcm_DifferenceAverage	− 0.879	0.385

### Inferential description of the selected features

**Fig. 4:**
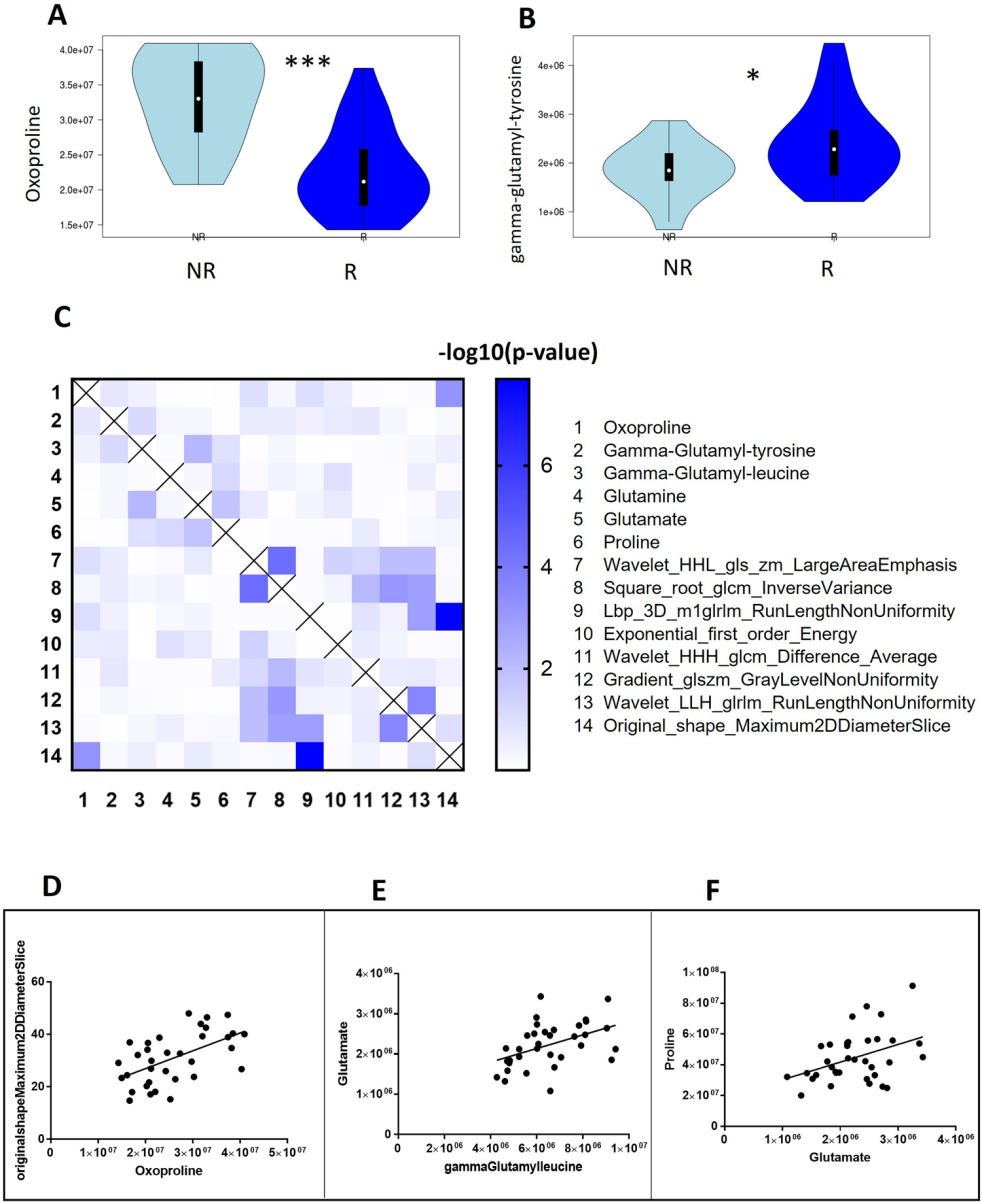
**A**, **B** Violin plot showing the distribution of oxoproline (Panel A) and gamma-glutamyl-tirosine (Panel B) for non-responder (in red) and responder (in green) patients. **C** Heatmap of the *p*-value for the correlation of 14 radiometabolomics features described in the legend on the right. **D** Pearson correlation between oxoproline levels and radiomics features called (original shape maximum 2D diameter slice). **E** Pearson correlation between gamma-glutamyl-leucine and glutamate levels. **F** Pearson correlation between glutamate and proline level. *** means *p*-value of Mann–Whitney test < 0.001, * means *p*-value of Mann–Whitney test < 0.05

**Fig. 5:**
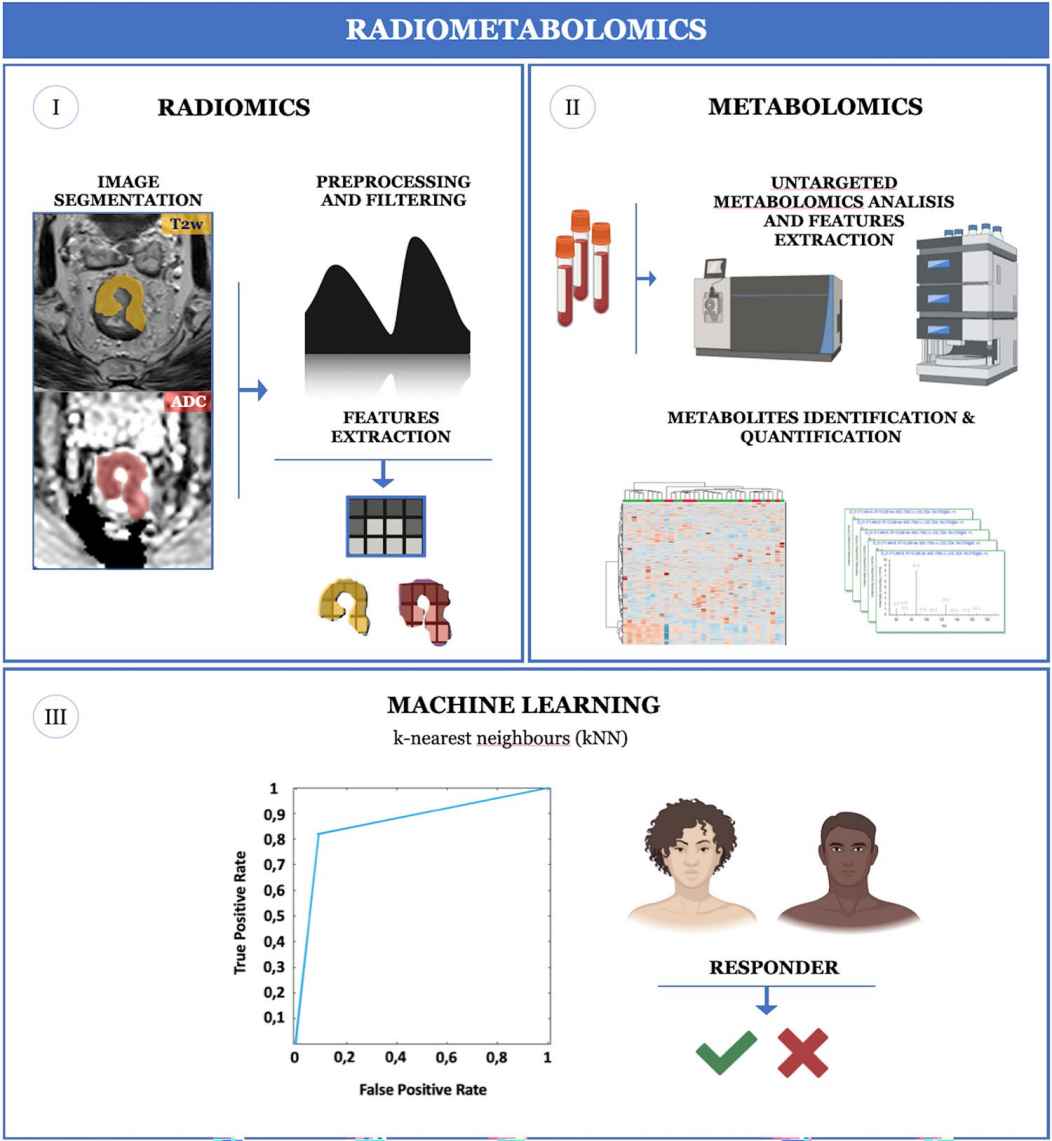
Study workflow. In the first step (I), rectal cancer was manually segmented on MR images (T2w, ADC), followed by radiomics features extraction. In the second step (II), plasma untargeted metabolomics analysis and metabolites identification and quantification were performed. In the final step (III), a machine learning algorithm (k-nearest neighbors—kNN) was used to select radiometabolomics features and ROC analysis delivered the classification model accuracy for treatment response prediction (created with BioRender.com)

Oxoproline levels were significantly (*p*-value = 0.0001) higher in NR patients (Fig. 4A). Interestingly, oxoproline is considered an oxidative stress marker, involved in the synthesis and degradation of glutathione in the glutathione cycle [44]. In this context, in addition to oxoproline, the gamma-glutamyl amino acids, glutamate, glutamine and proline are also involved. Thus, we extrapolated some of these identified and quantified molecules from the metabolomics data. Among all, gamma-glutamyl-tyrosine levels were significantly higher (*p*-value = 0.04) in responding patients before starting CRT (Fig. 4B). Furthermore, to integrate the data obtained from metabolomics and radiomics, we created a correlation matrix between the metabolites involved in the degradation and synthesis of glutathione’s cycle (numbers 1 to 6 of Fig. 4C legend) along with the main radiomics features (numbers 7–14 of Fig. 4C legend). Pearson correlation coefficients (*r*) were transformed into − log10 (*p*-value) and plotted as a Heatmap (Fig. 4C). R-coefficients and *p*-values calculated for each correlation are provided in Appendix 2 and 3 (Supplementary Material). As shown in the heatmap, significant correlations were found between the same metabolites and radiomics features. A significant moderate correlation was found between oxoproline levels and radiomics features “original shape maximum 2D diameter slice” with *r* = 0.557 and *p*-value = 0.0006 (Fig. 4D). Moreover, a significant fair correlation was observed between gamma-glutamyl-leucine and glutamate levels (*r* = 0.453 and *p*-value = 0.007), and between glutamate and proline levels (*r* = 0.405 and *p*-value = 0.001), as shown in Fig. 4 Panels E–F, respectively (Fig. 5).

## Discussion

Our study provided initial evidence that an MRI-based “radiometabolomic” approach has the potential to accurately predict treatment response of patients with LARC with high accuracy and at an early stage. This innovative approach, based on the integration of two -omics methods, could be transferred to the clinic in future, thus improving the patient selection for the most appropriate treatment. More in detail, adding radiometabolomics features to standard T2w conventional clinical features significantly improved the prognostic model. This work confirmed the preliminary results of recent radiomics studies on rectal cancer. Delli Pizzi et al. recently reported that a pre-treatment MRI-based radiomics ML model accurately predicts the treatment response in patients with LARC [15]. Similarly, Shin et al. presented a T2w and ADC-based radiomics model showing better classification performance than radiologists for diagnosing complete response after the completion of CRT [45]. Wang et al. reported that a radiomics model using pre-treatment radiotherapy planning CT images can predict treatment response and survival outcomes in LARC patients [46]. In our study, the predictive radiomics features were extracted from 1 mm slice thickness. In this regard, the last European Society of Gastrointestinal and Abdominal Imaging (ESGAR) consensus recommended that the slice thickness of the axial T2w image should be equal or inferior to 3 mm [4]. The predictive features of our study were mainly focused on tumor heterogeneity. For instance, tumors with a high degree of texture homogeneity were more likely associated with good treatment response. This result is in line with several previous non-rectal cancer studies showing that homogeneous texture features are associated with better clinical prognosis and neoadjuvant CRT response [14, 47, 48].

Our results suggested that treatment response prediction improves when imaging-based radiomics is combined with metabolomics. Metabolomics is an emerging field dedicated to the study of metabolites, their composition, interactions, dynamics and responses to diseases or therapies in cells, tissues and biofluids. The application of untargeted metabolomic analysis on peripheral blood may provide novel biomarkers of cancer treatment response [49]. Recently, Yang et al. investigated postoperative esophageal tissue via an untargeted metabolomics approach hypothesizing glycerophospholipids metabolism as a potential therapeutic target of tumor progression [50]. Moreover, several studies have recently explored metabolomics approaches in different fields of oncology, such as triple-negative breast cancer, gastric cancer and lung cancer [51–53]. For CRC, Brezmes et al. explored urinary NMR-based metabolomics to find novel biomarkers [54]. In addition, metabolic profile investigation associated with gut microbiome composition was recently applied to investigate potential diagnostic markers of individuals with CRC compared to healthy controls [55].

In our study, serum metabolic signature before treatment is significantly correlated to a down-regulation of the vitality of cancer cells in R compared to NR, highlighting how the metabolic study of serum before starting therapy can provide valuable information on outcome. Contextually, the expression of specific serum metabolites in R has highlighted a significant implication in oxidative stress response. Oxoproline accumulation coupled with low levels of gamma-glutamyl-tyrosine in NR patients may suggest a dysregulation in glutathione degradation and therefore a worse response to oxidative stress [56]. In our study, oxoproline levels were correlated with the tumor’s maximum diameter of the tumor measured on axial MR images (Fig. 4C).

This is, to our knowledge, the first study investigating the potential synergistic role of radiomics and metabolomics in rectal cancer, a translational research field we named “radiometabolomics.” A multi-disciplinary approach involving radiologists, oncologists, biochemists, radiation therapists and bioinformatics may support clinicians in selecting the most appropriate treatment tailored for each patient. This study aims to point out a proof-of-concept experimental workflow combining multi-omics features to explore new possible and pioneer strategies that, once overcome some limitations, could be ready to be moved into clinical practices. Firstly, the sample size is limited and obtained through a single-center retrospective study. Of note, in our work the implementation of the leave-one-out nCV minimized the effect of the reduced number of samples while avoiding overfitting [30]. It was also coupled with a robust feature selection approach (wrapper method), hence obtaining the maximum classification performance achievable with the available data no bias and good generalization. Because of the absence of analysis bias, an increase in the training sample size is expected to only improve the classification performance. Nonetheless, future studies, possibly prospective and multicenter, are needed to corroborate our findings and to obtain standardized multivariate models that can be routinely used in the clinical practice. Secondly, our 3 T MR scanner was scheduled over time and during the study for software upgrades which might have modified image quality. However, these changes did not cause changes in main MRI protocol parameters. Thirdly, we only used T2w imaging and DWI, without considering dynamic contrast-enhanced imaging (DCE). Nonetheless, the role of DCE in the primary staging of rectal cancer is controversial. In fact, according to the recent ESGAR consensus meeting, the use of DCE-MRI is not routinely recommended [4].

## Conclusion

Multi-omics staging has the potential to predict CRT response in patients with LARC, thus enhancing the predictive value of standard MRI and helping to avoid unnecessary surgical treatment. The proposed radiometabolomics integrated approach is into an embryonic phase which may encourage and promote deeper investigation in larger, prospective studies to push toward a better definition of the radiometabolomics role in personalized rectal cancer care.

### Supplementary Information

Below is the link to the electronic supplementary material.

Supplementary file1 (DOCX 25 KB)

## Data Availability

The datasets generated during and/or analyzed during the current study are not publicly available due to the clinical and confidential nature of the material. However, they can be made available from the corresponding author on reasonable request.
