# Supplementary material for: Multi-omics staging of locally advanced rectal cancer predicts treatment response: a pilot study
Source: Radiol Med. 2024 Mar 27;129(5):712–26. doi: 10.1007/s11547-024-01811-0 (PMC11088547; doi:10.1007/s11547-024-01811-0)
Supplement: Supplementary file 1 — Supplementary file1 (DOCX 25 KB) [file 11547_2024_1811_MOESM1_ESM.docx]

Appendix 1. MRI protocol parameters.

|  | **T2-Weighted**  **Turbo Spin Echo*** | **Diffusion-Weighted MRI**** |
| --- | --- | --- |
| Repetition time (msec) | 3000-5000 | 4500 |
| Echo time (msec) | 80 | 80 |
| Section thickness (mm) | 3 | 3 |
| Section gap (mm) | 0 | 0 |
| Acquisition Matrix Size | 188x167 | 68x66 |
| No. of signals acquired | 2 | 2 |
| Field of view (mm) | 150x150 | 200x200 |
| Sensitivity Encoding (SENSE) | Yes | No |
| Acquisition Time (min) | 2.39 | 6.03 |
| No. of sections | 30 | 30 |
| *= MR parameters are referred to axial T2-weighted images.  **=including b=1000 s/mm^2^ and Apparent Diffusion Coefficient (ADC) map | | |

Appendix 2. p-value of the Correlation matrix between metabolomics and radiomics features

|  | **Oxoproline** | **γ-Glutamyl-tyrosine** | **γ -Glutamyl-leucine** | **Glutamine** | **Glutamate** | **Proline** | **Wavele tHHL glszm Large Area Emphasis** | **Square Root Glcm Inverse Variance** | **Lbp 3D m1 glrlm Run Length Non Uniformity** | **Expo first order Energy** | **Wavelet HHH glcm Difference Average** | **Gradient glszm Gray Level Non Uniformity** | **Wavelet LLH glrlm Run Length Non Uniformity** | **Original shape Maximum 2D Diameter Slice** |
| --- | --- | --- | --- | --- | --- | --- | --- | --- | --- | --- | --- | --- | --- | --- |
| Oxoproline |  | 0.18 | 0.37 | 0.91 | 0.98 | 0.89 | 0.10 | 0.56 | 0.09 | 0.28 | 0.84 | 0.90 | 0.75 | 0.0006 |
| Gamma-Glutamyl-tyrosine | 0.18 |  | 0.07 | 0.54 | 0.55 | 0.89 | 0.26 | 0.25 | 0.36 | 0.24 | 0.17 | 0.53 | 0.98 | 0.63 |
| Gamma-Glutamyl-leucine | 0.37 | 0.07 |  | 0.77 | 0.007 | 0.11 | 0.92 | 0.54 | 0.98 | 0.99 | 0.83 | 0.74 | 0.55 | 0.32 |
| Glutamine | 0.91 | 0.54 | 0.77 |  | 0.57 | 0.06 | 0.69 | 0.42 | 0.58 | 0.12 | 0.83 | 0.86 | 0.31 | 0.78 |
| Glutamate | 0.98 | 0.55 | 0.007 | 0.57 |  | 0.01 | 0.22 | 0.89 | 0.47 | 0.23 | 0.83 | 0.91 | 0.66 | 0.26 |
| Proline | 0.89 | 0.89 | 0.11 | 0.06 | 0.01 |  | 0.95 | 0.83 | 0.86 | 0.82 | 0.25 | 0.88 | 0.60 | 0.50 |
| Wavelet HHL glszm Large Area Emphasis | 0.10 | 0.26 | 0.92 | 0.69 | 0.22 | 0.95 |  | 4.13E-05 | 0.78 | 0.04 | 0.06 | 0.01 | 0.01 | 0.69 |
| Square root glcm Inverse Variance | 0.56 | 0.25 | 0.54 | 0.42 | 0.89 | 0.83 | 4.13E-05 |  | 0.73 | 0.49 | 0.008 | 0.0007 | 0.001 | 0.64 |
| Lbp 3Dm1 glrlm Run Length Non Uniformity | 0.09 | 0.36 | 0.98 | 0.58 | 0.47 | 0.86 | 0.78 | 0.73 |  | 0.97 | 0.15 | 0.37 | 0.001 | 1.98E-08 |
| Exponential first order Energy | 0.28 | 0.24 | 0.99 | 0.12 | 0.23 | 0.82 | 0.04 | 0.49 | 0.97 |  | 0.26 | 0.47 | 0.82 | 0.70 |
| Wavelet HHH glcm Difference Average | 0.84 | 0.17 | 0.83 | 0.83 | 0.83 | 0.25 | 0.06 | 0.008 | 0.15 | 0.26 |  | 0.27 | 0.24 | 0.39 |
| Gradient glszm Gray Level Non Uniformity | 0.90 | 0.53 | 0.74 | 0.86 | 0.91 | 0.88 | 0.01 | 0.0007 | 0.37 | 0.47 | 0.27 |  | 0.0002 | 0.81 |
| Wavelet LLH glrlm Run Length Non Uniformity | 0.75 | 0.98 | 0.55 | 0.31 | 0.66 | 0.60 | 0.01 | 0.001 | 0.001343 | 0.82 | 0.24 | 0.0002 |  | 0.10 |
| Original shape Maximum 2D Diameter Slice | 0.0006 | 0.63 | 0.34 | 0.78 | 0.26 | 0.50 | 0.69 | 0.64 | 1.98E-08 | 0.70 | 0.39 | 0.81 | 0.10 |  |

Appendix 3. r-coefficients of the Correlation matrix between metabolomics and radiomics features

|  | **Oxoproline** | **γ-Glutamyl-tyrosine** | **γ -Glutamyl-leucine** | **Glutamine** | **Glutamate** | **Proline** | **Wavele tHHL glszm Large Area Emphasis** | **Square Root Glcm Inverse Variance** | **Lbp 3D m1 glrlm Run Length Non Uniformity** | **Expo first order Energy** | **Wavelet HHH glcm Difference Average** | **Gradient glszm Gray Level Non Uniformity** | **Wavelet LLH glrlm Run Length Non Uniformity** | **Original shape Maximum 2D Diameter Slice** |
| --- | --- | --- | --- | --- | --- | --- | --- | --- | --- | --- | --- | --- | --- | --- |
| **Oxoproline** |  | -0.22 | -0.18 | -0.09 | 0.04 | -0.03 | 0.16 | -0.18 | 0.3 | -0.4 | -0.04 | 0.13 | -0.01 | 0.55 |
| **Gamma-Glutamyl-tyrosine** | -0.22 |  | 0.21 | 0.15 | 0.06 | 0.14 | 0.17 | -0.15 | -0.23 | -0.18 | 0.18 | 0.16 | -0.15 | -0.12 |
| **Gamma-Glutamyl-leucine** | -0.18 | 0.21 |  | 0.14 | 0.47 | 0.25 | -0.1 | -0.06 | 0.12 | 0.05 | -0.07 | 0.02 | -0.01 | 0.18 |
| **Glutamine** | -0.09 | 0.15 | 0.14 |  | 0.13 | 0.2 | 0.07 | -0.16 | -0.09 | -0.28 | -0.09 | 0.15 | -0.15 | -0.1 |
| **Glutamate** | 0.04 | 0.06 | 0.47 | 0.13 |  | 0.34 | -0.15 | 0.04 | 0.21 | 0.21 | 0.14 | 0.06 | 0.1 | 0.22 |
| **Proline** | -0.03 | 0.14 | 0.25 | 0.2 | 0.34 |  | -0.05 | -0.04 | 0.13 | -0.06 | -0.14 | 0.11 | -0.03 | 0.13 |
| **Wavelet HHL glszm Large Area Emphasis** | 0.16 | 0.17 | -0.1 | 0.07 | -0.15 | -0.05 |  | -0.83 | -0.03 | -0.53 | 0.24 | 0.75 | -0.6 | -0.02 |
| **Square root glcm Inverse Variance** | -0.18 | -0.15 | -0.06 | -0.16 | 0.04 | -0.04 | -0.83 |  | -0.14 | 0.47 | -0.19 | -0.77 | 0.6 | -0.13 |
| **Lbp 3Dm1 glrlm Run Length Non Uniformity** | 0.3 | -0.23 | 0.12 | -0.09 | 0.21 | 0.13 | -0.03 | -0.14 |  | 0.003 | 0.22 | 0.005 | 0.4 | 0.85 |
| **Exponential first order Energy** | -0.4 | -0.18 | 0.05 | -0.28 | 0.21 | -0.06 | -0.53 | 0.47 | 0.003 |  | -0.25 | -0.38 | 0.22 | -0.11 |
| **Wavelet HHH glcm Difference Average** | -0.04 | 0.18 | -0.07 | -0.09 | 0.14 | -0.14 | 0.24 | -0.19 | 0.22 | -0.25 |  | 0.04 | 0.29 | 0.12 |
| **Gradient glszm Gray Level Non Uniformity** | 0.13 | 0.16 | 0.02 | 0.15 | 0.06 | 0.11 | 0.75 | -0.77 | 0.005 | -0.38 | 0.04 |  | -0.7 | 0.07 |
| **Wavelet LLH glrlm Run Length Non Uniformity** | -0.01 | -0.15 | -0.01 | -0.15 | 0.1 | -0.03 | -0.6 | 0.6 | 0.4 | 0.22 | 0.29 | -0.7 |  | 0.29 |
| **Original shape Maximum 2D Diameter Slice** | 0.55 | -0.12 | 0.18 | -0.1 | 0.22 | 0.13 | -0.02 | -0.13 | 0.85 | -0.11 | 0.12 | 0.07 | 0.29 |  |
